# Supplementary material for: cfDNA Methylation Profiles and T-Cell Differentiation in Women with Endometrial Polyps
Source: Cells. 2022 Dec 9;11(24):3989. doi: 10.3390/cells11243989 (PMC9777338; doi:10.3390/cells11243989)
Supplement: Supplementary file 1 [file cells-11-03989-s001.zip › cells-2026049-supplementary.pdf]

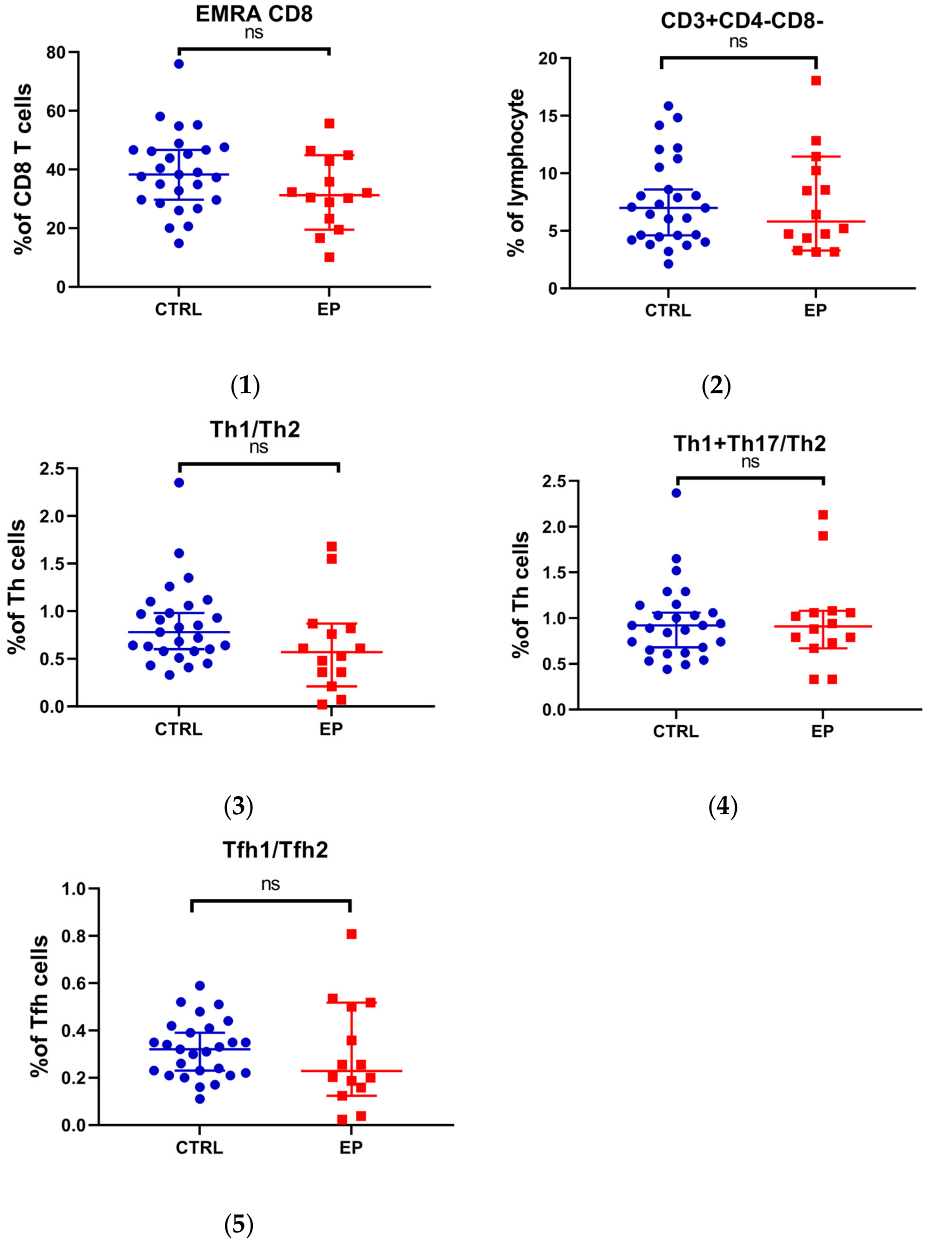


**Supplementary Figure S1.** Comparison of the ratio of EMRA CD8+ T cells, DN T cells, Th1/Th2, Th1+Th17/Th2, and Tfh1/Tfh2 between EP patients and healthy controls. (1) Percentage of EMRA CD8+ T cells in CD8+ T cells; (2) Percentage of DN T cells in lymphocyte; (3) Ratio of Th1/Th2 in Th cells; (4) Ratio of Th1+Th17/Th2 in Th cells; (5) Ratio of Tfh1/Tfh2 in Tfh cells. ns, not significant.

**Supplementary Table S1.** The primers for multiplex amplicon PCR.

| Gene | Forward Primers | Reverse Primers | Products |
|---|---|---|---|
| IGF1R | TTGAAAATTTAAGGAGGTTATTAGGAA | TCTATTTACTA-AAATATATTCATCCTT | 114 |
| CTBP1 | TGTTGTAATTTYGTT-GTGG | TCTAACTAAAAACCCAACATTATCAA | 121 |

| | | | |
|---|---|---|---|
| TCF7L1 | TTGTTAAGGAAAAAGTGGTTT | CCAAC-CTATAAACAATCAAAAACTAATA | 136 |
| E2F3 | GGTAGGTTAG-TTTATAAATTGTTTT | TCTTCCTTTACCCTTAAAAAT | 162 |
| CACNA2D4 | AGAAGGTAGYG-AAGTTGGTTATTA | CCCCTAA-TACTACTACTACTACT-AAACCT | 109 |
| KCNJ12 | TGGAAGGGAT-TAAATGAATTAG | ACAAAAAC-TATTCAACTAACTAAAA | 130 |
| TPO-1 | TGATTAGTTAGGATA-TATAAGAGGTT | CTTCCAAAAACTTAATTACCCACC | 158 |
| TPO-2 | TTTGAGGAA-TAAAGTAA-TATTGTTAG | AAACTCCTCAAACTTTCCTA | 95 |
| UGT1A8/10-1 | TTTTATGTGTGTGTTTATTGTTGA | CCCTAAAAA-TAAATTTCTCCACCA | 123 |
| UGT1A8/10-2 | AGAGGTTATTAGGTGGTGGTT | ATTAAA-TATAACCCAACAAAAA | 136 |
| CABP5 | GGTTTTTGGGTAG-TGAGATGG | AAAACAAATAC-CATACTCTTAATCAC | 149 |
| CST9 | TGGGTTTTTTGAGA-GAGAGG | CCTACTCTTAATTTCCTCAACC | 136 |
| ITGA2 | TTTTTGAAATTTGGTT-GATT | TAAATTATCTAAAA-TACTCACCTACTTACT | 139 |
| DLGAP2 | TTTTGAGAGTT-GAAGTTTTATA-GAAAGG | ATCTACTAAA-TAAAAAAAAAC-CTTTAACAT | 142 |
| ESPNP | GAGAA-TATTGTGGTTAG-TTTTTTG | CTCCTACCTC-TACATAAACAC-CAAATC | 138 |
| NBPF25P | AGGG-GAAGTTTTTAAGGGG | ACCCAACAAAAAATACAAACCCTA | 150 |
| RASA3-1 | ATTATGTTTAGYGG-TAATATGTTTA | CATCTATTAAACTCACATCCTCACC | 109 |
| RASA3-2 | TATTTTGGTGATA-TAGAGGTTGGA | AAAATTATCTTATAACCAATCTTCA | 145 |
| ZIM2 | GGTGGGTTTTAGGTTTATTAG | TAAAAAAAC-CACCCCAAAAA | 107 |

| | | | |
|---|---|---|---|
| PXDN | GTATTGGTTTTTAATT TTAGAAGGG | AAACCTATAC-CAAACTCATAAAAA | 100 |
| HDAC4 | AGGGGGTGTTATGA-TAAAATTGAA | CTAAC-CTAACAAAACCCATCCC | 126 |
| VAV2 | TAAATGTATGA-TAAAGATA-TAGGTAGAGGG | AAACAAAAAAATCAAATAAACTCTC | 139 |

**Supplementary Table S2.** Basic information of 19 DMGs and related DMCs in this study.

| Gene | Location | chr | CpG site position |
|---|---|---|---|
| IGF1R | genebody | chr15 | 99334874 |
| IGF1R | genebody | chr15 | 99334911 |
| CTBP1 | promoter | chr4 | 1243970 |
| CTBP1 | promoter | chr4 | 1243978 |
| CTBP1 | promoter | chr4 | 1243980 |
| CTBP1 | promoter | chr4 | 1244006 |
| CTBP1 | promoter | chr4 | 1244017 |
| CTBP1 | promoter | chr4 | 1244042 |
| TCF7L1 | genebody | chr2 | 85476406 |
| TCF7L1 | genebody | chr2 | 85476431 |
| TCF7L1 | genebody | chr2 | 85476438 |
| E2F3 | genebody | chr6 | 20480045 |
| E2F3 | genebody | chr6 | 20480051 |
| E2F3 | genebody | chr6 | 20480059 |
| E2F3 | genebody | chr6 | 20480083 |
| E2F3 | genebody | chr6 | 20480106 |
| CACNA2D4 | genebody | chr12 | 1974772 |
| CACNA2D4 | genebody | chr12 | 1974788 |
| CACNA2D4 | genebody | chr12 | 1974794 |
| CACNA2D4 | genebody | chr12 | 1974799 |
| CACNA2D4 | genebody | chr12 | 1974810 |
| KCNJ12 | genebody | chr17 | 21314267 |
| KCNJ12 | genebody | chr17 | 21314274 |
| TPO | promoter | chr2 | 1417106 |
| TPO | promoter | chr2 | 1417109 |
| TPO | promoter | chr2 | 1417146 |
| TPO | promoter | chr2 | 1417153 |
| TPO | genebody | chr2 | 1426786 |
| TPO | genebody | chr2 | 1426812 |
| UGT1A8/UGT1A10 | genebody | chr2 | 234545237 |
| UGT1A8/UGT1A10 | genebody | chr2 | 234545296 |

| | | | |
|---|---|---|---|
| UGT1A8/UGT1A10 | genebody | chr2 | 234621873 |
| UGT1A8/UGT1A10 | genebody | chr2 | 234621898 |
| UGT1A8/UGT1A10 | genebody | chr2 | 234621908 |
| CABP5 | promoter | chr19 | 48547989 |
| CABP5 | promoter | chr19 | 48548040 |
| CABP5 | promoter | chr19 | 48548046 |
| CST9 | promoter | chr20 | 23587041 |
| CST9 | promoter | chr20 | 23587062 |
| CST9 | promoter | chr20 | 23587090 |
| CST9 | promoter | chr20 | 23587099 |
| ITGA2 | promoter | chr5 | 52283720 |
| ITGA2 | promoter | chr5 | 52283760 |
| DLGAP2 | promoter | chr8 | 1153482 |
| DLGAP2 | promoter | chr8 | 1153495 |
| DLGAP2 | promoter | chr8 | 1153524 |
| ESPNP | genebody | chr1 | 17023331 |
| ESPNP | genebody | chr1 | 17023334 |
| ESPNP | genebody | chr1 | 17023384 |
| ESPNP | genebody | chr1 | 17023391 |
| NBPF25P | genebody | chr1 | 148853834 |
| NBPF25P | genebody | chr1 | 148853852 |
| NBPF25P | genebody | chr1 | 148853878 |
| RASA3 | genebody | chr13 | 114880761 |
| ZIM2 | genebody | chr19 | 57306584 |
| ZIM2 | genebody | chr19 | 57306612 |
| ZIM2 | genebody | chr19 | 57306631 |
| ZIM2 | genebody | chr19 | 57306640 |
| PXDN | genebody | chr2 | 1654109 |
| PXDN | genebody | chr2 | 1654127 |
| PXDN | genebody | chr2 | 1654131 |
| HDAC4 | genebody | chr2 | 240212933 |
| HDAC4 | genebody | chr2 | 240212943 |
| HDAC4 | genebody | chr2 | 240212960 |
| VAV2 | genebody | chr9 | 136726437 |
| VAV2 | genebody | chr9 | 136726465 |
| VAV2 | genebody | chr9 | 136726477 |
| VAV2 | genebody | chr9 | 136726484 |

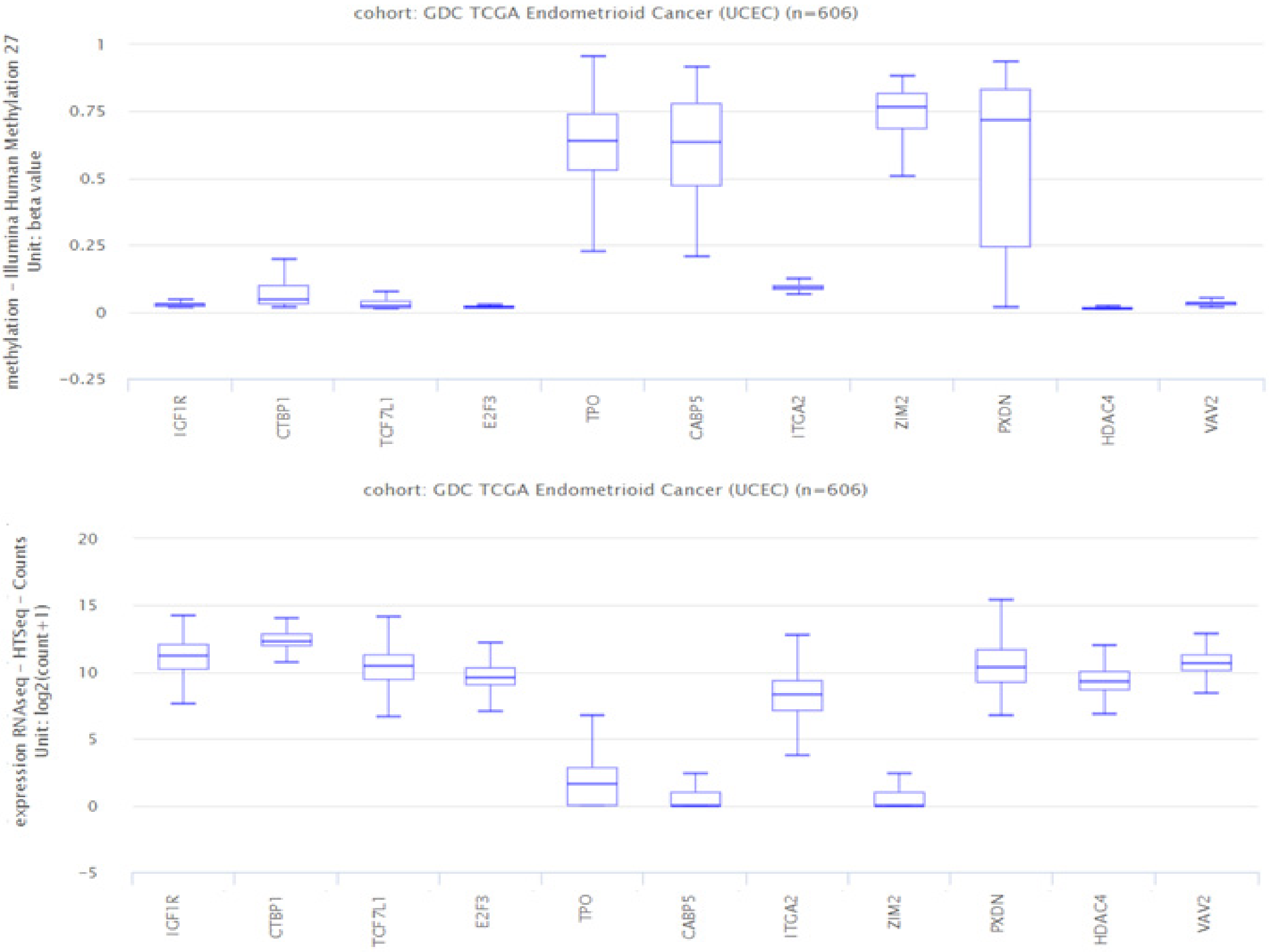


**Supplementary Figure S2.** The methylation and expression levels of *IGF1R*, *CTBP1*, *TCF7L1*, *E2F3*, *ITGA2*, *HDAC4*, *TPO*, *CABP5*, *ZIM2*, *PXDN*, and *VAV2* genes are found in the GDC TCGA Endometrioid Cancer cohort (UCEC, n=606).
